# Aryloxypropanolamine targets amyloid aggregates and reverses Alzheimer-like phenotypes in Alzheimer mouse models

**DOI:** 10.1186/s13195-022-01112-6

**Published:** 2022-11-29

**Authors:** Hee Yang Lee, Soljee Yoon, Jeong Hwa Lee, Keunwan Park, Youngeun Jung, Illhwan Cho, Donghee Lee, Jisu Shin, Kyeonghwan Kim, Sunmi Kim, Jimin Kim, Koeun Kim, Seung Hoon Han, Seong Muk Kim, Hye Ju Kim, Hye Yun Kim, Ikyon Kim, Young Soo Kim

**Affiliations:** 1 Department of Pharmacy and Yonsei Institute of Pharmaceutical Sciences, College of Pharmacy, Yonsei University, Yeonsu-gu, Incheon, 21983 South Korea; 2 Department of Integrative Biotechnology & Translational Medicine, Yonsei University, Yeonsu-gu, Incheon, 21983 South Korea; 3 Natural Product Informatics Research Center, Korea Institute of Science and Technology (KIST), Gangwon-do, 25451 South Korea; 4 Amyloid Solution, Bundang-gu, Seongnam-si, Gyeonggi-do 13486 South Korea

**Keywords:** Alzheimer’s disease, Amyloid-β, Drug discovery, Tau, Small molecule

## Abstract

**Background:**

Aggregated amyloid-β (Aβ) is considered a pathogenic initiator of Alzheimer’s disease (AD), in strong association with tau hyperphosphorylation, neuroinflammation, synaptic dysfunction, and cognitive decline. As the removal of amyloid burden from AD patient brains by antibodies has shown therapeutic potential, the development of small molecule drugs inducing chemical dissociation and clearance of Aβ is compelling as a therapeutic strategy. In this study, we synthesized and screened aryloxypropanolamine derivatives and identified 1-(3-(2,4-di-tert-pentylphenoxy)-2-hydroxypropyl)pyrrolidin-1-ium chloride, YIAD002, as a strong dissociator of Aβ aggregates.

**Methods:**

The dissociative activity of aryloxypropanolamine derivatives against Aβ aggregates were evaluated through in vitro assays. Immunohistochemical staining, immunoblot assays, and the Morris water maze were used to assess the anti-Alzheimer potential in YIAD002-treated 5XFAD and transgenic APP/PS1 mice. Target-ligand interaction mechanism was characterized via a combination of peptide mapping, fluorescence dissociation assays, and constrained docking simulations.

**Results:**

Among 11 aryloxypropanolamine derivatives, YIAD002 exerted strongest dissociative activity against β-sheet-rich Aβ aggregates. Upon oral administration, YIAD002 substantially reduced amyloid burden and accordingly, improved cognitive performance in the Morris water maze and attenuated major pathological hallmarks of AD including tauopathy, neuroinflammation, and synaptic protein loss. Mechanism studies suggest that YIAD002 interferes with intermolecular β-sheet fibrillation by directly interacting with KLVFFA and IGLMVG domains of Aβ. In addition, YIAD002 was found to possess dissociative activity against aggregates of pyroglutamate-modified Aβ and tau.

**Conclusions:**

Collectively, our results evince the potential of chemical-driven dissociation of Aβ aggregates by aryloxypropanolamines as a therapeutic modality of the amyloid clearance approach.

**Supplementary Information:**

The online version contains supplementary material available at 10.1186/s13195-022-01112-6.

## Background

Aggregation and accumulation of amyloid-β (Aβ) in the brain of Alzheimer’s disease (AD) patients is considered a primary hallmark and initial pathological event of disease progression [1]. Generated by the proteolytic cleavage of the amyloid precursor protein (APP), monomeric Aβ spontaneously aggregates into toxic oligomers and β-sheet-rich fibrils that trigger downstream events such as the hyperphosphorylation of tau, neuroinflammation, synaptic dysfunction, and eventually cognitive decline [2]. Disassembly and removal of Aβ aggregates from the brain have been investigated as AD therapeutic strategies. However, the highly transient and polymorphic nature of Aβ aggregation has impeded the rational design of Aβ-targeting drugs [3], limiting current disease-modifying treatments to immunotherapy [4]. The next expected direction in the AD drug pipeline is a chemical drug modality of amyloid clearance, as small molecules have bypassed amyloid-related imaging abnormalities [5], high blood-brain barrier (BBB) permeability, oral bioavailability, and production/cost-efficiency [6]. Through phenotypic anti-Aβ screening of small molecules, we previously demonstrated chemical-driven reduction of Aβ plaques in the brains of transgenic AD animal models [7–9].

Aryloxypropanolamines are a class of compounds that are clinically used for a wide array of pharmacological activities including antidepressant and anticancer activities [10]. Compared to the majority of compounds in this class with a secondary amine moiety, biological functions of compounds bearing a tertiary amine group have been less studied. Furthermore, the therapeutic potential of aryloxypropanolamines against AD pathology have not been established. Thus, a small set of 11 aryloxypropanolamine-based small molecules possessing a tertiary amine were newly synthesized for biological evaluation. By screening for anti-amyloid activity, we identified 1-(3-(2,4-di-tert-pentylphenoxy)-2-hydroxypropyl)pyrrolidin-1-ium chloride, YIAD002, as a strong dissociator of Aβ oligomers and fibrils. Upon oral administration of YIAD002, we observed the clearance of amyloid plaques in two different AD transgenic mouse models, with subsequent amelioration of cognitive behavior in the Morris water maze and alleviation of downstream pathology. We then identified the site of dissociation by YIAD002 through a set of peptide mapping assays and predicted target-ligand binding conformation via docking simulations on multiple in silico models of Aβ. Furthermore, we discovered that YIAD002 has dissociative activity against aggregates of the pyroglutamate-modified Aβ, Aβ(pE3-42), and the microtubule-binding repeat domain of tau, suggesting that the dual targeting of Aβ and tau may contribute to enhanced therapeutic effects in vivo.

## Methods

### Study design

In vitro anti-Aβ screening assays utilizing Thioflavin T (ThT) fluorescence were performed to identify small molecules that could dissociate aggregates of Aβ. Animal studies using 5XFAD and APP/PS1 transgenic AD mouse models were performed to assess amyloid burden reduction, amelioration of AD pathology, and cognitive improvement. BBB-specific in vitro parallel artificial membrane permeability assay (PAMPA) and ADME assessments were performed to assess druggability. Fragment-based peptide mapping assays and constrained docking simulations predicted target-ligand binding conformations.

### Chemical syntheses of small molecules

Unless specified, all reagents and starting materials were purchased from commercial sources and used as received without purification. “Concentrated” refers to the removal of volatile solvents via distillation using a rotary evaporator. “Dried” refers to pouring onto, or passing through, anhydrous magnesium sulfate followed by filtration. Flash chromatography was performed using silica gel (230–400 mesh) with hexanes, ethyl acetate, and dichloromethane as the eluents. All reactions were monitored by thin-layer chromatography on 0.25-mm silica plates (F-254) visualized with UV light. Melting points were measured using a capillary melting point apparatus. ^1^H and ^13^C NMR spectra were recorded on a 400 MHz NMR spectrometer and were described as chemical shifts, multiplicity (s, singlet; d, doublet; t, triplet; q, quartet; m, multiplet), coupling constant in hertz (Hz), and number of protons. HRMS were measured with an electrospray ionization (ESI) and Q-TOF mass analyzer.

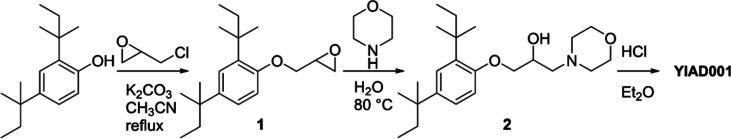



### General procedure for the synthesis of YIAD001

To a stirred solution of epichlorohydrin (1.3 equiv) in acetonitrile (7 mL) were added 2,4-di-*tert*-amylphenol (2.13 mmol, 1 equiv) and K_2_CO_3_ (2.0 equiv) at room temperature. After being stirred at reflux for 24 h (monitored by TLC), the reaction mixture was concentrated under reduced pressure, extracted with dichloromethane (3 × 5 mL), and washed with water. The organic layer was dried over MgSO_4_ and concentrated *in vacuo* to afford the crude product which was used for the next step without further purification. To a mixture of the crude residue **1** in water (7 mL) was added morpholine (1.5 equiv) at room temperature. After being stirred at 80°C for 16 h (monitored by TLC), the reaction mixture was extracted with ethyl acetate (3 × 5 mL) and washed with water. The organic layer was dried over MgSO_4_ and concentrated *in vacuo* to afford the crude product which was purified by silica gel column chromatography (hexane/EtOAc/dichloromethane = 3:1:1) to give 2 (692.6 mg, 86%). Then, 1M HCl in Et_2_O (2 mL) was added to a solution of 2 in Et_2_O (10 mL) on ice to give a salt form of 2, YIAD001. Characterization data of the newly synthesized small molecules are listed as follows:

***4-(3-(2,4-Di-tert-pentylphenoxy)-2-hydroxypropyl)morpholin-4-ium chloride (YIAD001)***

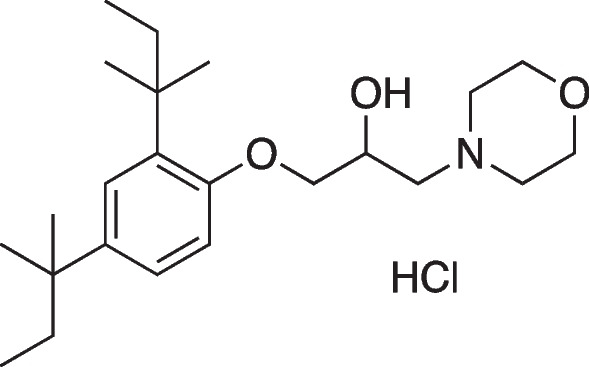



White solid, mp: 192.5–193.1°C; ^1^H NMR (400 MHz, CDCl_3_) δ 12.29 (s, 1H), 7.17 (s, 1H), 7.10 (d, *J* = 8.4 Hz, 1H), 6.76 (d, *J* = 7.6 Hz, 1H), 5.39 (s, 1H), 4.69–4.79 (m, 1H), 4.26–4.39 (m, 2H), 4.09–4.15 (m, 1H), 3.94–4.04 (m, 3H), 3.67–3.76 (m, 2H), 3.24–3.36 (m, 2H), 3.00–3.11 (m, 1H), 2.89–2.99 (m, 1H), 1.72–1.80 (m, 2H), 1.55–1.62 (m, 2H), 1.33 (s, 6H), 1.25 (s, 6H), 0.60–0.68 (m, 6H); ^13^C NMR (100 MHz, CDCl_3_) δ 154.1, 141.8, 134.9, 126.1, 124.3, 111.4, 69.0, 64.1, 63.7, 63.6, 54.7, 52.9, 38.5, 37.4, 37.0, 33.8, 28.5, 28.1, 28.1, 9.5, 9.1; HRMS (ESI-QTOF) *m/z* [M+Na]^+^ calcd for C_23_H_39_NNaO_3_ 400.2822, found 400.2825.

***1-(3-(2,4-Di-tert-pentylphenoxy)-2-hydroxypropyl)pyrrolidin-1-ium chloride (YIAD002)***

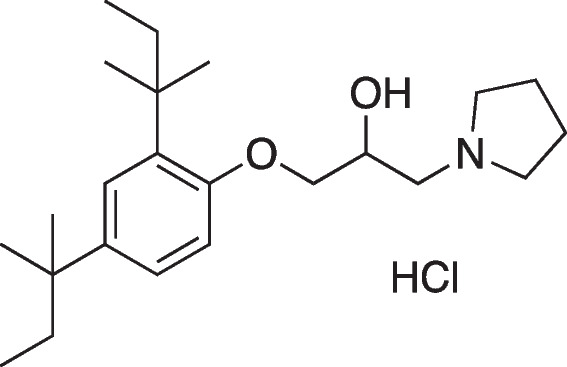



White solid, mp: 179.8–180.7°C; ^1^H NMR (400 MHz, CDCl_3_) δ 11.78 (s, 1H), 7.16 (s, 1H), 7.09 (dd, *J* = 2.0, 8.4 Hz, 1H) 6.76 (d, *J* = 8.4 Hz, 1H) 5.41 (s, 1H), 4.54–4.63 (m, 1H), 4.09–4.14 (m, 1H), 3.99–4.05 (m, 1H), 3.93–3.99 (m, 2H), 3.35–3.41 (m, 2H), 2.93–30.1 (m, 1H), 2.83–2.91 (m, 1H), 2.21–2.30 (m, 2H), 2.05–2.14 (m, 2H), 1.71–1.81 (m, 2H), 1.55–1.62 (m, 2H), 1.32 (s, 6H), 1.24 (s, 6H), 0.59–0.66 (m, 6H); ^13^C NMR (100 MHz, CDCl_3_) δ 154.2, 141.6, 134.8, 126.0, 124.3, 111.4, 69.1, 65.8, 61.1, 55.9, 54.4, 38.5, 37.4, 37.0, 33.7, 28.5, 28.1, 28.0, 23.3, 23.1, 9.5, 9.1; HRMS (ESI-QTOF) *m/z* [M+H]^+^ calcd for C_23_H_40_NO_2_ 362.3054, found 362.3061.

***4-(2-Hydroxy-3-(4-methoxyphenoxy)propyl)morpholin-4-ium chloride (YIAD003)***

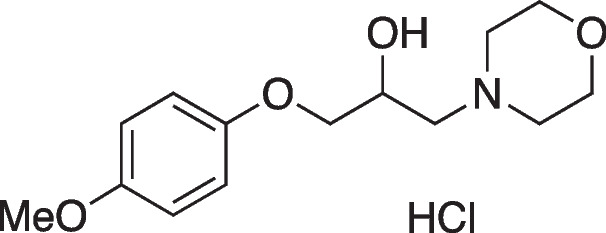



White solid, mp: 160.2–160.5°C; ^1^H NMR (400 MHz, DMSO-d_6_) δ 10.76 (s, 1H), 6.85–6.99 (m, 4H), 5.98 (s, 1H), 4.42 (s, 1H), 3.83–4.01 (s, 6H), 3.73 (s, 3H), 3.44–3.60 (m, 2H), 3.10–3.36 (m, 4H); ^13^C NMR (100 MHz, DMSO-d_6_) δ 154.0, 152.7, 116.0, 115.0, 71.1, 64.0, 63.5, 59.5, 55.8, 55.8; HRMS (ESI-QTOF) *m/z* [M+H]^+^ calcd for C_14_H_22_NO_4_ 268.1543, found 268.1551.

***1-(2-Hydroxy-3-(4-methoxyphenoxy)propyl)piperidin-1-ium chloride (YIAD004)***

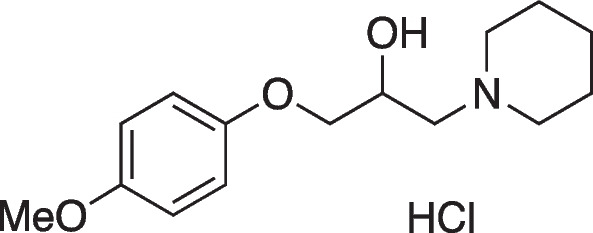



Pale yellow solid, mp: 169.3–169.9°C; ^1^H NMR (400 MHz, CDCl_3_) δ 6.75–6.84 (m, 4H), 4.52–4.65 (m, 1H), 4.04–4.11 (m, 1H), 3.82–3.89 (m, 1H), 3.75 (s, 3H), 2.86–3.50 (m, 6H), 1.90–2.20 (m, 4H), 1.46–1.80 (m, 2H); ^13^C NMR (100 MHz, CDCl_3_) δ 154.3, 152.0, 115.4, 114.7, 69.6, 64.2, 62.6, 55.7, 55.6, 22.8, 21.9; HRMS (ESI-QTOF) *m/z* [M+Na]^+^ calcd for C_15_H_23_NNaO_3_ 288.1570, found 288.1571.

***4-(3-(4-Bromophenoxy)-2-hydroxypropyl)morpholin-4-ium chloride (YIAD005)***

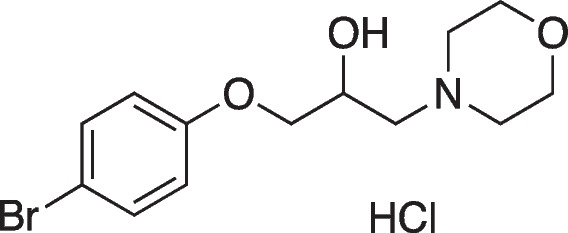



White solid, mp: 172.1–172.7°C; ^1^H NMR (400 MHz, CDCl_3_) δ 12.11 (s, 1H), 7.37 (d, *J* = 7.6 Hz, 2H), 6.76 (d, *J* = 7.6 Hz, 2H), 5.39 (s, 1H), 4.72 (s, 1H), 4.21–4.36 (m, 2H), 4.00–4.13 (m, 2H), 3.86–3.99 (m, 2H), 3.62–3.81 (m, 2H), 3.23–3.34 (m, 2H), 2.90–3.12 (m, 2H); ^13^C NMR (100 MHz, CDCl_3_) δ 156.9, 132.5, 116.2, 113.9, 69.3, 63.9, 63.7, 62.5, 54.6, 52.9; HRMS (ESI-QTOF) *m/z* [M+H]^+^ calcd for C_13_H_19_BrNO_3_ 316.0543, found 316.0539.

***1-(3-(4-Bromophenoxy)-2-hydroxypropyl)piperidin-1-ium chloride (YIAD006)***

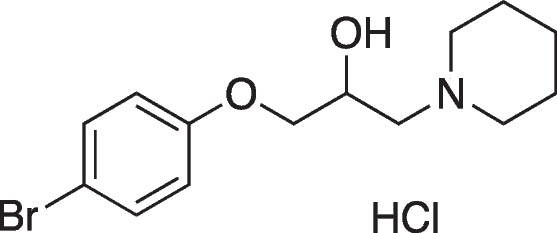



Pale yellow solid, mp: 178.1–178.6°C; ^1^H NMR (400 MHz, CDCl_3_) δ 11.28 (s, 1H), 7.36 (d, *J* = 7.6 Hz, 2H), 6.76 (d, *J* = 8.4 Hz, 2H), 5.59 (s, 1H), 4.58–4.70 (m, 1H), 4.06–4.14 (m, 1H), 3.84–3.93 (m, 1H), 3.64–3.76 (m, 2H), 3.11–3.32 (m, 2H), 2.65–2.90 (m, 2H), 2.17–2.40 (m, 2H), 1.86–1.94 (m, 2H), 1.75–1.86 (m, 1H), 1.35–1.53 (m, 1H); ^13^C NMR (100 MHz, CDCl_3_) δ 157.0, 132.4, 116.2, 113.7, 69.2, 64.1, 62.5, 56.2, 54.3, 22.7, 21.9; HRMS (ESI-QTOF) *m/z* [M+H]^+^ calcd for C_14_H_21_BrNO_2_ 314.0750, found 314.0761.

***1-(3-(4-Bromophenoxy)-2-hydroxypropyl)pyrrolidin-1-ium chloride (YIAD007)***

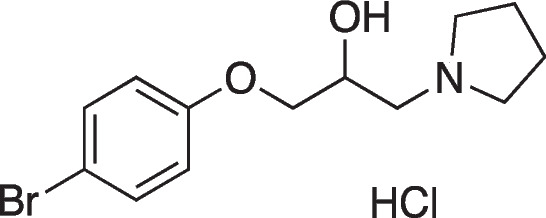



Pale green solid, mp: 176.9–177.7°C; ^1^H NMR (400 MHz, CDCl_3_) δ 11.70 (s, 1H), 7.37 (d, *J* = 6.8 Hz, 2H), 6.77 (d, *J* = 5.6 Hz, 2H), 4.44–4.70 (m, 1H), 4.07–4.17 (m, 1H), 3.86–4.03 (m, 3H), 3.22–3.47 (m, 2H), 2.73–3.13 (m, 2H), 2.20–2.32 (m, 2H), 2.04–2.18 (m, 2H); ^13^C NMR (100 MHz, CDCl_3_) δ 157.0, 132.5, 116.3, 113.8, 69.3, 65.5, 60.3, 56.0, 54.7, 23.4, 23.2; HRMS (ESI-QTOF) *m/z* [M+H]^+^ calcd for C_13_H_19_BrNO_2_ 300.0594, found 300.0606.

***1-(3-(4-(Tert-butyl)phenoxy)-2-hydroxypropyl)piperidin-1-ium chloride (YIAD008)***

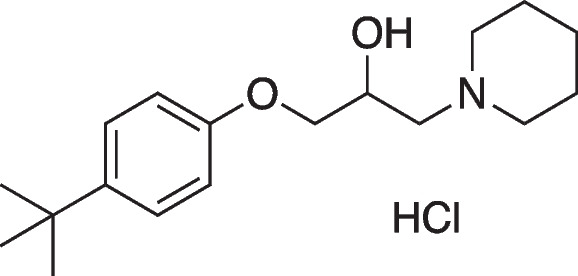



White solid, mp: 201.7–202.2°C; ^1^H NMR (400 MHz, CDCl_3_) δ 11.32 (s, 1H), z7.29 (d, *J* = 8.8 Hz, 2H), 6.81 (d, *J* = 8.8 Hz, 2H), 4.55–4.70 (m, 1H), 4.09–4.18 (m, 1H), 3.88 (t, *J* = 8.8 Hz, 1H), 3.70 (t, *J* = 13.8 Hz, 2H), 3.21–3.31 (m, 1H), 3.09–3.20 (m, 1H), 2.66–2.90 (m, 2H), 2.20–2.40 (m, 2H), 1.81–1.95 (m, 3H), 1.39–1.50 (m, 1H), 1.28 (s, 9H); ^13^C NMR (100 MHz, CDCl_3_) δ 155.6, 144.3, 126.4, 113.9, 68.8, 64.2, 63.0, 56.3, 54.2, 34.1, 31.5, 22.6, 21.9; HRMS (ESI-QTOF) *m/z* [M+H]^+^ calcd for C_18_H_30_NO_2_ 292.2271, found 292.2262.

***4-(3-(4-(Tert-butyl)phenoxy)-2-hydroxypropyl)morpholin-4-ium chloride (YIAD009)***

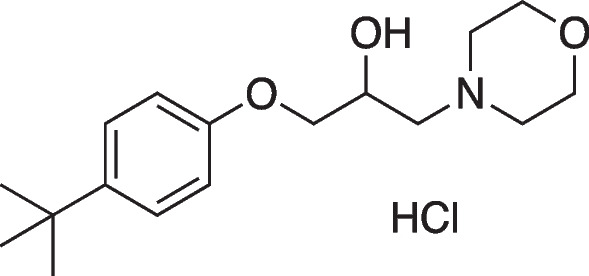



White solid, mp: 197.9–198.3°C; ^1^H NMR (400 MHz, CDCl_3_) δ 12.20 (s, 1H), 7.30 (d, *J* = 8.8 Hz, 2H), 6.81 (d, *J* = 8.4 Hz, 2H), 5.29 (s, 1H), 4.80(s, 1H), 4.25–4.37 (m, 2H), 4.09–4.18 (m, 1H), 3.99 (t, *J* = 11.8 Hz, 2H), 3.87–3.94 (m, 1H), 3.69 (t, *J* = 11.0 Hz, 2H), 3.20–3.35 (m, 2H), 2.91–3.11 (m, 2H), 1.29 (s, 9H); ^13^C NMR (100 MHz, CDCl_3_) δ 155.5, 144.4, 126.4, 113.9, 68.9, 64.0, 63.7, 63.7, 62.9, 54.5, 52.8, 34.1, 31.5; HRMS (ESI-QTOF) *m/z* [M+H]^+^ calcd for C_17_H_28_NO_3_ 294.2064, found 294.2066.

***1-(3-(4-(Tert-butyl)phenoxy)-2-hydroxypropyl)pyrrolidin-1-ium chloride (YIAD010)***

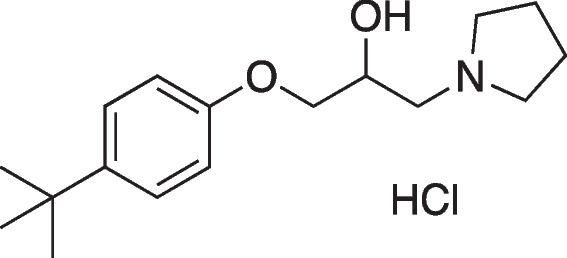



Pale yellow solid, mp: 139.9–140.4°C; ^1^H NMR (400 MHz, CDCl_3_) δ 7.30 (d, *J* = 7.6 Hz, 2H), 6.82 (d, *J* = 7.6 Hz, 2H), 5.30 (s, 1H), 4.55 (s, 1H), 4.09–4.18 (m, 1H), 3.83–4.00 (m, 2H), 3.24–3.44 (m, 2H), 1.98–2.32 (m, 4H), 1.57–1.81 (m, 2H), 1.29 (s, 9H); ^13^C NMR (100 MHz, CDCl_3_) δ 155.6, 144.3, 126.4, 113.9, 68.8, 65.6, 60.5, 34.1, 31.5, 23.3; HRMS (ESI-QTOF) *m/z* [M+Na]^+^ calcd for C_17_H_27_NNaO_2_ 300.1934, found 300.1939.

***1-(2-Hydroxy-3-phenoxypropyl)piperidin-1-ium chloride (YIAD011)***

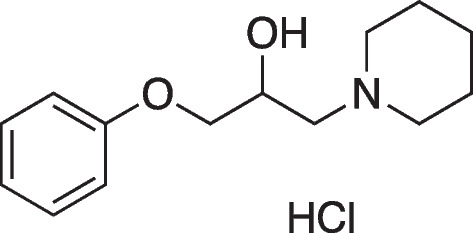



Pale yellow solid, mp: 151.2–151.9°C; ^1^H NMR (400 MHz, CDCl_3_) δ 7.26–7.32 (m, 2H), 6.97 (t, *J* = 7.4 Hz, 1H), 6.88 (d, *J* = 8.0 Hz, 2H), 4.54–4.64 (m, 1H), 4.10–4.17 (m, 1H), 3.91 (t, *J* = 8.6 Hz, 1H), 2.94–3.37 (m, 6H), 1.95–2.11 (m, 4H), 1.51–1.76 (m, 2H); ^13^C NMR (100 MHz, CDCl_3_) δ 158.0, 129.6, 121.4, 114.4, 68.9, 64.3, 62.6, 55.1, 23.0, 22.1; HRMS (ESI-QTOF) *m/z* [M+H]^+^ calcd for C_14_H_22_NO_2_ 236.1645, found 236.1624.

### Preparation of Aβ peptides and tau fragments

Synthesized monomeric Aβ(1–42), DAEFRHDSGY EVHHQKLVFF AEDVGSNKGA IIGLMVGGVV IA, Aβ(1–40), DAEFRHDSGY EVHHQKLVFF AEDVGSNKGA IIGLMVGGVV, Aβ(pE3-42), pEFRHDSGYEV HHQKLVFFAE DVGSNKGAII LMVGGVVIA, Tau(244–274) repeat 1 (R1), QTAPVPMPDL KNVKSKIGST ENLKHQPGGG K, Tau(275–305) R2, VQIINKKLDL SNVQSKCGSK DNIKHVPGGG S, Tau(306–336) R3, VQIVYKPVDL SKVTSKCGSL GNIHHKPGGG Q, and Tau(337–368) R4, VEVKSEKLDF KDRVQSKIGS LDNITHVPGG GN, were acquired by Fmoc solid phase peptide synthesis as previously described [11]. Recombinant tau K18 fragments, QTAPVPMPDL KNVKSKIGST ENLKHQPGGG KVQIINKKLD LSNVQSKCGS KDNIKHVPGG GSVQIVYKPV DLSKVTSKCG SLGNIHHKPG GGQVEVKSEK LDFKDRVQSK IGSLDNITHV PGGGNKKIE, cloned from full-length human (hTau40) were purified from *Escherichia coli* BL21 (DE3) cells [12, 13].

### ThT fluorescence assay

ThT fluorescence was used to assess the in vitro inhibition of Aβ(1–42) and disaggregation of Aβ(1–42), Aβ(1–40), Aβ(pE3-42), recombinant tau K18, and tau repeats R1, R2, R3, R4 by YIAD compounds. In house synthetic Aβ(1–42) was dissolved in DMSO (1 mM, 100% DMSO) and diluted to 20-fold in deionized water to make 50 μM Aβ(1–42) stocks (5% DMSO). YIAD compounds were first dissolved in DMSO (100 mM, 100% DMSO) and serially diluted with deionized water to make YIAD stocks with concentrations of 1, 10, 100, and 1000 μM (10% DMSO). For the Aβ(1–42) inhibition assay, monomeric Aβ(1–42) stocks and YIAD stocks were co-incubated at 37°C for 3 days. For Aβ(1–42) disaggregation assay, monomeric Aβ(1–42) stocks were preincubated at 37°C for three days to form Aβ(1–42) aggregates, and subsequently, YIAD stocks were added for an additional 3 days. For Aβ(1-40) disaggregation assays, Aβ(1–40) (50 μM, 5% DMSO) was preincubated at 37°C for 3 days to induce aggregation, and co-incubated with YIAD001 and YIAD002 for an additional 3 days. For Aβ(pE3-42) disaggregation assays, in house synthetic Aβ(pE3-42) (50 μM, 5% DMSO) was preincubated at 37°C for 1 day and co-incubated with YIAD002 for an additional 3 days. For tau K18 disaggregation assay, recombinant tau K18 fragments (35 μM) were dissolved in PBS with 0.1 mg/mL of heparin and 100 μM of DTT, then pre-aggregated at 37°C for 3 days. Then, YIAD stocks were added to aggregated K18 fragments for an additional 3 days. For tau repeat disaggregation assays, tau repeats (R1, R2, R3, R4) (35 μM) were dissolved in PBS with 0.1 mg/mL of heparin and 100 μM of DTT, then pre-aggregated at 37°C for 1 day. Then, YIAD002 was added to aggregated repeats for an additional 3 days. Prior to fluorescence intensity measurement, ThT solution (5 μM) was prepared by dissolving ThT, purchased from Sigma-Aldrich (MO, USA) in 50 mM glycine buffer (pH 8.5). To quantify the effect of inhibition and disaggregation, 25 μL of all samples and 75 μL of ThT solution were loaded into a half-area 96-well black plate in triplicate. Fluorescence intensity was measured at 450 (excitation) and 485 (emission) using the TECAN Infinite 200 PRO microplate reader.

### SDS-PAGE with PICUP and silver staining

SDS-PAGE with photo-induced cross-linking of unmodified proteins (PICUP) and silver staining was used to evaluate the disaggregating effects of YIAD001 and YIAD002 by visualizing the size distributions and band intensity of Aβ(1–42). Samples were prepared under the same conditions as the Aβ(1–42) disaggregation ThT fluorescence assay. Amyloid proteins in the samples from the aforementioned disaggregation assay were subjected to cross-linking, initiated by irradiation (three exposures, each for 1 second) in the presence of tris(2,2′-bipyridyl)dichlororuthenium(II) hexahydrate (Ru(Bpy)) (1 mM) and 1 μL of ammonium persulfate (20 mM). Reactions were immediately quenched with β-mercaptoethanol and boiled for 5 min at 95°C. After cross-linking the samples, we separated amyloid species by SDS-PAGE on a 15% tris-tricine gel and performed silver staining according to the PlusOne Silver Staining kit protocol (GE Healthcare, USA).

### BBB-PAMPA

Commercially available Double-Sink PAMPA (Pion® Double sink) assay was performed using a two compartment 96-well microtiter plate and protocols specified by the manufacturer. Progesterone and lidocaine were used as positive controls and ranitidine was used as a negative control. Stock solutions of controls and YIAD002 were dissolved in DMSO (10 mM) and subsequently diluted to 200-fold in pION buffer (pH 7.4) to 50 μM. The donor plate of the PAMPA sandwich was loaded with 50 μM solutions, and after wetting the artificial membrane with BBB lipid solution, the acceptor plate was loaded with acceptor sink buffer. Then, the acceptor plate was placed on the donor plate and incubated at 25°C for 4 h. Compound concentration in the reference, donor, and acceptor plates were measured on a UV plate.

### CYP450 inhibition assay

Incubation mixtures for Cytochrome P450s (CYPs) inhibition assay contained human liver microsomes (0.25 mg/ml), 0.1 M phosphate buffer (pH 7.4), 10 μM of YIAD002, and an enzyme substrate cocktail (phenacetin 50 μM, diclofenac 10 μM, S-mephenytoin 100 μM, dextromethorphan 5 μM, and midazolam 2.5 μM). The mixture was pre-incubated at 37°C for 5 min and 1 mM NAPDH was added to initiate reactions. After 15 min of incubation, acetonitrile with terfenadine was added to stop reactions and microsomal solutions were centrifuged at 14,000 rpm at 4°C for 5 min. For LC-MS/MS analysis, supernatants were injected on a Kinetex C18 column (2.1 × 100 mm, 2.6 μm particle size, Phenomenex, USA). The composition of the mobile phase was 0.1% formic acid in water (A) and 0.1% formic acid in acetonitrile (B).

### Plasma stability

To assess the plasma stability of YIAD002, YIAD002 stock was dissolved at a concentration of 40 μM in DMSO. 5 μL of YIAD002 stock was added to 195 μL of human plasma or 195 μL of rat plasma and incubated at 37°C for the following time points: 0, 30, and 120 min. At the end of incubation, acetonitrile with chlorpropamide was added to stop reactions. The solutions were centrifuged at 14,000 rpm at 4°C for 5 min and the supernatant was analyzed with LC-MS/MS. High performance liquid chromatography was carried out on a Kinetex C18 column (2.1 × 100 mm, 2.6 μm particle size, Phenomenex, USA). The composition of the mobile phase was 0.1% formic acid in water (A) and 0.1% formic acid in acetonitrile (B).

### Microsomal stability

To evaluate the rate of drug metabolism, we tested YIAD002 stability in liver microsomes of human, rat, and mouse. Microsomal concentration was 0.5 mg/ml in 0.1 M phosphate buffer (pH 7.4). 1 μM of YIAD002 was added to microsome solutions and was prewarmed at 37°C for 5 min. NADPH-regenerating solution was added to microsomes and incubated at 37°C for 30 min. At the end of incubation, acetonitrile with chlorpropamide was added to stop reactions and microsomal solutions were centrifuged at 14,000 rpm at 4°C for 5 min. For LC-MS/MS analysis, supernatants were injected on a Kinetex C18 column (2.1 × 100 mm, 2.6 μm particle size, Phenomenex, USA). The composition of the mobile phase was 0.1% formic acid in water (A) and 0.1% formic acid in acetonitrile (B).

### Animals

5XFAD mice (strain name: B6SJL-Tg(APPSwFlLon,PSEN1*M146L*L286V)6799Vas/-Mmjax) expressing the Swedish (K670N/M671L), Florida (I716V), and London (V717I) mutations in APP and the M146L and L286V mutations in PSEN1) and wild-type mice (C57BL/6 x SJL) were originally obtained from the Jackson Laboratory (Bar Harbor, ME, USA). Heterozygous transgenic mice were maintained through cross-breeding with the wild-type mice, and the genotype of all mice was confirmed via PCR analysis of tail DNA following the standard PCR condition from the Jackson Laboratory. All mice were housed in a laboratory animal room and were maintained under controlled temperature, humidity, and an alternating 12-h light–dark cycle. Access to food and water were available ad libitum. Protocols were approved by Institutional Animal Care and Use Committee (IACUC) of the Yonsei Laboratory Animal Research Center.

APP/PS1 (strain name: B6C3-Tg(APPswe,PSEN1dE9)85Dbo/Mmjax) expressing chimeric mouse/human amyloid precursor protein (Mo/HuAPP695swe) and a mutant human presenilin 1 (PS1-dE9) were obtained from the Jackson Laboratory (Bar Harbor, ME, USA). All mice were housed in a laboratory animal room and were maintained under controlled temperature, humidity, and an alternating 12-h light–dark cycle. Access to food and water were available ad libitum. Protocols were approved by the IACUC of NDIC Inc. (Kyunggido, Korea; approval No. P191114).

### Oral administration of YIAD001 and YIAD002 to 5XFAD mice

To test the in vivo efficacy of YIAD001 and YIAD002, female 5XFAD mice (*n* = 25) and female wildtype mice (*n* = 7) were assessed at the age of 4.5 months. YIAD compounds were dissolved in DMSO and diluted in drinking water to a final concentration of 50 mg/kg/day (5% DMSO, 5% Tween) for oral administration. The three compounds, YIAD001, YIAD002, and YIAD003, were freely administered via drinking water to 4.5-month-old 5XFAD mice (*n* = 6 for each group) for 5 weeks. As a control, 5% DMSO, 5% Tween in drinking water was provided ad libitum to littermate 5XFAD mice (*n* = 7) and wildtype mice (*n* = 7) for 5 weeks.

After the administration period, the mice were deeply anaesthetized with 4% avertin through intraperitoneal injection and transcardially perfused with 0.9% saline. Subsequently, the mice were sacrificed for brain excision. Each collected brain was divided through the mid-sagittal section; the hippocampal and cortical regions were dissected from one half and stored at −80°C for subsequent brain lysates, while the other half was immersed in 4% paraformaldehyde (pH 7.4) for 24 h and 30% sucrose for 48 h at 4°C and subsequently deep-froze at −80°C for immunohistochemistry.

### Oral administration of YIAD002 to APP/PS1 mice

To test whether YIAD002 could reduce amyloid plaques in another AD mouse model, APP/PS1 transgenic mice (*n* = 24) and wildtype mice (*n* = 5) were assessed at the age of 7.5 months. YIAD002 was dissolved in PBS (0.5% methylcellulose) at two different concentrations (10 and 30 mg/kg/day) and administrated daily to APP/PS1 mice (*n* = 8 for each group) via oral gavage for 8 weeks. As a control, vehicle solutions were orally administrated to littermate APP/PS1 mice (*n* = 8) and wildtype mice (*n* = 5).

### Morris water maze

The Morris water maze was used to evaluate whether YIAD002 could improve cognitive function in APP/PS1 mice during the tenth week of administration. A circular stainless pool with a radius of 90 cm and height of 50 cm with water kept at 22±1°C. Nontoxic opaque white paint was used to hide a platform with a radius of 9 cm hidden 1 cm beneath the surface. The training phase consisted of 5 days, in which the mice were given 60 s to find the platform. On circumstances where a mouse could not reach the platform within 60 s, it was placed on the platform where it had to remain for 10 s. The probe test took place on the sixth day, in which the platform was removed and the mice swam freely for 60 s. Swimming trajectories were recorded and analyzed using SMART VIDEO TRACKING Software (Panlab, USA). To assess cognitive improvement in YIAD002-treated mice groups, latency to target (seconds) and the number of target crossings were compared to vehicle-treated APP/PS1 mice. After the probe trial, the mice were sacrificed and the collected brains were fixed in formalin.

### Immunohistochemical staining

Coronal hippocampal sections of 5XFAD mice brains (35 μm) were cut with a Cryostat (Leica, CM1860) and mounted onto glass slides. Antigen retrieval was performed by submerging slides in 1% SDS and unspecific binding was blocked through incubation in 20% horse serum in PBS for 1 h at RT. Aβ deposits were visualized through immunolabeling by anti-6E10 antibody (1:200 in PBS with 5% horse serum, Biolegend 803003) and detection by fluorescent secondary antibody (1:200 in PBS, IgG Alexa 488). Brain sections were additionally stained with Hoechst 33342. Fluorescent images of the brain sections were taken on a Leica DM2500 fluorescence microscope. Amyloid plaques were quantified using Image-J software (NIH).

Formalin-fixed APP/PS1 mice brains were infiltrated with paraffin using a tissue processor (LEICA ASP300S, Germany). Blocks of tissues were produced with a tissue embedding center and sectioned into thin slices (4 μm), which were mounted onto glass slides. To prepare for 6E10 staining, slides were deparaffinized, rehydrated, and blocked with 0.03% H2O2 to remove endogenous peroxidase. For antigen retrieval, slides were boiled in Tris/EDTA solution (pH 9.0) using a pressure cooker. Unspecific binding was blocked through incubation in 4% BSA at RT for 30 min. Slides were immunolabeled with anti-6E10 antibody (1:2000, Novus Biologicals NBP2-62566) at RT for 1 h and HRP-labeled secondary antibody (Dako K4003) for 30 min and subsequently stained with DAB (3,3′-Diaminobenzidine) and counterstained with Mayer’s hematoxylin. Stained slides were scanned using ZEISS Axio scan.Z1 microscope. Amyloid plaques were quantified using Image-J software (NIH).

### Lysis of 5XFAD mouse brain tissues

Isolated cortical and hippocampal tissue samples were homogenized in ice-cold RIPA buffer with protease inhibitor cocktail and phosphatase inhibitor cocktail. Lysed tissues were incubated in ice for 30 min and subsequently centrifuged at 14,000 rpm at 4°C for 30 min. The supernatants of the brain lysates were collected and soluble protein concentrations were quantified with the Pierce BCA protein assay kit (Thermo Fisher Scientific, USA).

### Dot blot assay

To analyze total and oligomeric amyloid levels, protein samples (12 μg in 2 μL) and prepared in vitro disaggregation assay samples (2 μL) were directly blotted on nitrocellulose membranes. After blocking with 5% skim milk in TBS-T, the membranes were probed with anti-6E10 antibody (1:1,000, Biolegend 803003) and anti-A11 antibody (1:2000, Invitrogen AHB0052) at 4°C overnight. HRP-conjugated goat anti-mouse antibody (1:10,000, Bethyl Laboratories A90-116P) and anti-rabbit secondary antibody (1:10,000, Bethyl Laboratories A120-101P) were used for 6E10 and A11, respectively. Proteins were detected using an ECL kit (Thermo Fisher Scientific, USA). Membranes were washed with TBS-T in between steps, three times for 10 min.

### Molecular weight cut-off filtration of soluble lysates

To exclusively investigate soluble Aβ levels in cortical lysates, guanidinium hydrochloride (GdnHCl) in a lysis buffer (5.0 M GdnHCl, 50 mM Tris-HCl, pH 8.0) was utilized to solubilize and denature endogenous Aβ aggregates prior to filtration. The homogenates were incubated with agitation for 3 h at RT. The incubated samples were then quantified to prepare protein samples (10 μg in 4 μL) and were filtered through a 100 kDa molecular weight cut-off (MWCO) filter, centrifuged at 14,000 rpm at RT for 30 min, to remove APP. For subsequent dot blot assay, total 8 μL of the filtrate was spotted onto a nitrocellulose membrane and probed with anti-6E10 antibody (1:1000, Biolegend 803003).

### Western blot

Western blot analysis was performed to assess YIAD002-induced alterations of protein biomarkers in 5XFAD mice brain lysates. Twenty micrograms of lysate samples were loaded onto 12% tris-tricine gels, separated by SDS-PAGE, and transferred to nitrocellulose membranes. Membranes were blocked by 5% skim milk or 5% BSA in TBS-T. The following primary antibodies were used for immunoblotting: anti-6E10 for APP (1:1000, Biolegend 803003), anti-AT8 (1:1000, Invitrogen MN1020), anti-Tau (1:1000, Santa Cruz SC390476), anti-ionized calcium-binding adapter molecule (Iba1) (1:1000, CST, 17198S), anti-glial fibrillar acidic protein (GFAP) (1:2000, Milipore AB5541), anti-postsynaptic density protein 95 (PSD95) (1:1000, CST 3450S), anti-synaptophysin (1:1000, Milipore MAB5258), and anti-β-Actin (1:10,000, Milipore MAB1501). Membranes were incubated with primary antibodies at 4°C overnight and probed with HRP-conjugated IgG antibodies (1:10,000) in RT for an hour. Proteins were detected using an ECL kit (Thermo Fisher Scientific, USA). Membranes were washed with TBS-T in between steps, three times for 10 min.

### Mapping assay of Aβ(1–42)

Aβ fragments made up of six overlapping amino acid residues and a cysteine residue at the C-terminal were acquired by Fmoc solid phase peptide synthesis as previously described [11]. Full length Aβ(1–42) with an additional cysteine residue at the C-terminal was used for comparison. The peptides were dissolved in DMSO (1.5 M) and diluted in binding buffer (0.1 M sodium phosphate, 0.15 M sodium chloride, 10 mM EDTA; pH 7.2) to prepare 50 μg/mL peptide solutions. To immobilize the Aβ fragments and monomers to maleimide-activated microplates, 100 μL of peptide solutions (50 μg/mL) was added and incubated at RT for 2 h while shaking. Cysteine solution (10 μg/mL with binding buffer) was added to cap unreacted maleimide groups. Flamma 552-conjugated Aβ(1–42) was prepared in deionized water (10% DMSO) (10 μM) and incubated in the wells at 37°C for 2 h. To define the fluorescence baseline for each well, fluorescence intensity was measured at 555 (excitation) and 580 (emission). YIAD002 (500 μM in binding buffer, 7.5% DMSO) was added to each well and incubated at RT for 24 h while shaking. Fluorescence intensity was remeasured at 555 (excitation) and 580 (emission) and baseline fluorescence was subtracted to quantify the change in fluorescence intensity caused by YIAD002. The plate was washed with 200 μL of wash buffer (0.1 M sodium phosphate, 0.15 M sodium chloride, 0.05% Tween®-20 Detergent; pH 7.2) per well three times in between steps.

### Docking models against Aβ and tau

The three-dimensional conformers for YIAD002 were prepared and separately docked to the multiple Aβ and tau oligomer structures (2BEG, 5KK3, 2NAO, 2MXU for Aβ and 2V5B, 5V5C, 2ON9 for tau) representing structural polymorphism (Table S1). The conformer generation procedure resulted in 129 YIAD002 conformers. For each oligomer structure, the binding site was initially predicted by global docking search with PatchDock. In Aβ, the binding site was confined near the Aβ(16–21) and Aβ(32–37) fragments in edge strands of β-sheets. The top 50 docking conformations for each oligomer structure were used to estimate potential binding sites for the subsequent local docking refinement by Autodock vina. Finally, all docking conformations were pooled together for Aβ and tau, respectively, and the docking model that showed the lowest binding energy was selected for the structural analysis.

### Statistical analysis

Graphs were obtained with GraphPad Prism 7 and statistical analyses were performed with Student’s unpaired *t*-tests or one-way analysis of variance followed by Bonferroni’s post hoc comparisons (*P < 0.05, **P < 0.01, ***P < 0.001, ****P < 0.0001; other comparisons were not significant). The error bars represent the SEMs.

## Results

### Synthesis and screening of compounds dissociating Aβ aggregates in vitro

**Fig. 1:**
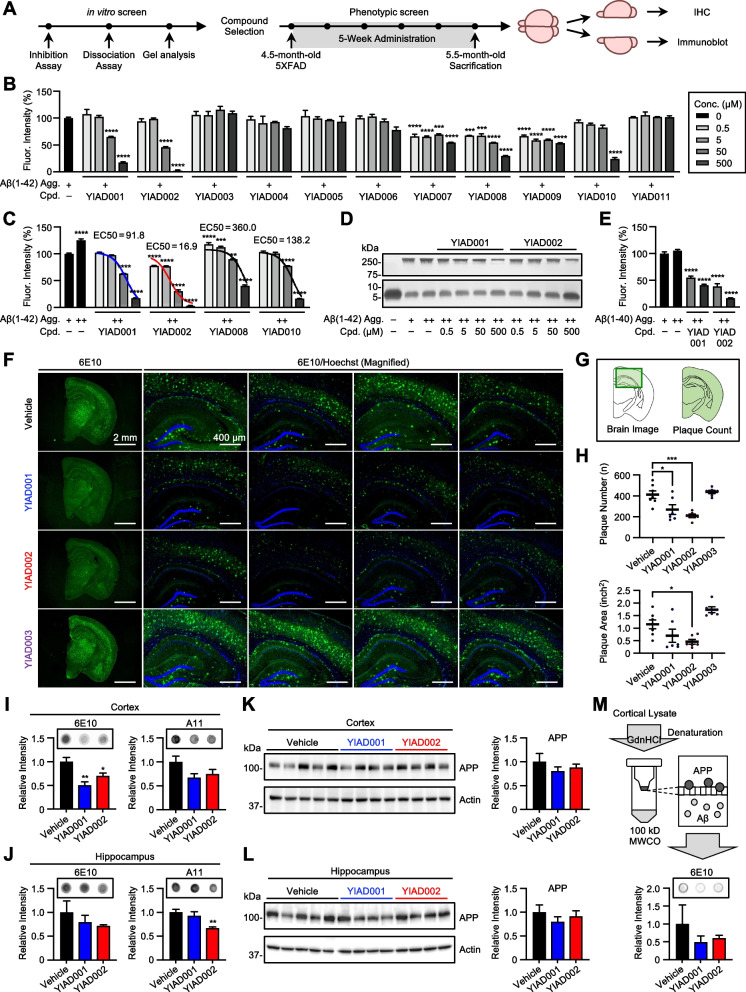
Screening aryloxypropanolamines derivatives for dissociative activity against Aβ aggregates. **A** Scheme of phenotypic screen for aryloxypropanolamines derivatives. **B** Inhibitory effects of synthesized compounds against Aβ(1–42) aggregation were evaluated using ThT fluorescence. **C** Dissociative effects of compounds. **D** Gel electrophoresis with PICUP and silver staining. **E** Disaggregation effects of YIAD001 and YIAD002 against Aβ(1–40) aggregates. Aggregation time for Aβ is indicated as “–” for 0 day, “+” for 3 days, and “++” for 6 days. All fluorescence intensities were normalized and statistically compared to Aβ(1–42)-only 3 days (+) control (100%). **F**–**M** YIAD001 (50 mg/kg/day, *n* = 6), YIAD002 (50 mg/kg/day, *n* = 6), and YIAD003 (50 mg/kg/day, *n* = 6) were orally administered to 4.5-month-old female 5XFAD mice for 5 weeks. **F** Brains were immunostained with 6E10 (green) and Hoechst (blue). **G** Illustration indicating the region for magnified brain image (left) and plaque count (right). **H** Plaque number and area of the whole brain of the mice were quantified. Dot blot assay using soluble fractions of cortical (**I**) and hippocampal (**J**) lysates with 6E10 and A11 antibodies. Immunoblot of soluble fractions of cortical (**K**) and hippocampal (**L**) lysates. Relative intensities of the blots were quantified in ratio to actin. **M** Schematic illustration and results of MWCO filtration and dot blot assay. Cortical lysates were denatured by GdnHCl and filtered through a 100 kDa MWCO filter to remove APP (100–140 kDa). All statistical comparisons were made with the normalized vehicle-treated 5XFAD mice. Full gels, brain images, and blot images are provided in additional files. One-way analysis of variance followed by Bonferroni’s post hoc comparisons tests were performed in all statistical analyses (*P < 0.05, **P < 0.01, ***P < 0.001, ****P < 0.0001). Data are presented as mean ± SEM. Abbreviations: Conc. = concentration, Agg. = aggregation, Cpd. = compound, Fluor. = fluorescent

Chemical inhibitors of Aβ aggregation were identified from 11 aryloxypropanolamines derivatives through a series of anti-Aβ screens utilizing the ThT fluorescent dye which detects β-sheet secondary structures (Fig. 1A) [14]. While Aβ(1–40) is the most abundant Aβ isoform, Aβ(1–42) is the most toxic and aggregation-prone isoform [15]. As an initial screen, compounds at concentrations of 0.5, 5, 50, and 500 μM were co-incubated with synthesized monomeric Aβ(1–42) (25 μM) at 37°C for 3 days. Fibrillation of Aβ(1–42) was inhibited by YIAD001 (82.55%, *P* < 0.0001), YIAD002 (98.15%, *P* < 0.0001), YIAD008 (70.69%, *P* < 0.0001), and YIAD010 (75.96%, *P* < 0.0001) (Fig. 1B). Next, the four compounds were tested for their ability to dissociate preformed aggregates of Aβ(1–42). Aggregated Aβ(1–42) was prepared by incubating monomeric Aβ(1–42) (25 μM) at 37°C for 3 days and the four compounds were subsequently added and co-incubated with the aggregates for an additional 3 days. Fibrillar aggregates were significantly decreased by the addition of YIAD001 (82.61%, *P* < 0.0001), YIAD002 (99.94%, *P* < 0.0001), YIAD008 (59.89%, *P* < 0.0001), and YIAD010 (83.56%, *P* < 0.0001) (Fig. 1C). The calculated EC_50_ values of dissociative activity against Aβ(1–42) by YIAD001, YIAD002, YIAD008, and YIAD010 were 91.8, 16.9, 360.0, and 138.2 μM, respectively (Fig. 1C). The two stronger dissociators, YIAD001 and YIAD002, were selected for further examination.

Partial dissociation of Aβ aggregates may unintentionally induce the formation of soluble oligomers, which are reported as the most toxic Aβ species [16]. To see whether YIAD001 and YIAD002 dissociate fibrils to monomeric states, the size distribution and intensity of Aβ(1–42) bands were visualized by silver staining of SDS-PAGE. Prior to gel electrophoresis, PICUP chemistry was performed to fix the metastable Aβ(1–42) assemblies [17]. In comparison to the non-aggregated Aβ(1–42) control, fibrillar content was markedly increased in the aggregated Aβ(1–42) controls (Fig. 1D). YIAD001 and YIAD002 reduced fibrils and increased monomers of aggregated Aβ(1–42) at 500 μM, indicating the dissociation by the compounds reverted fibrils back to monomeric forms (Fig. 1D). We confirmed that YIAD001 and YIAD002 dissociate Aβ(1–42) aggregates in a dot blot assay using oligomer-specific antibody A11 (Fig. S1).

As YIAD001 and YIAD002 show dissociative activity against Aβ(1–42), we next tested their dissociative effect against the Aβ(1–40) isoform. Aβ(1–40) (25 μM) was aggregated at 37°C for 3 days and YIAD001 and YIAD002 at the concentrations of 50 and 500 μM were co-incubated for an additional 3 days. At 500 μM, aggregates of Aβ(1–40) were significantly decreased by both YIAD001 (58.98%, *P* < 0.0001) and YIAD002 (83.35%, *P* < 0.0001) (Fig. 1E). Our results show that both compounds significantly dissociate aggregates of Aβ(1–42) and Aβ(1–40).

### YIAD001 and YIAD002 reduce amyloid burden in 5XFAD mice

We then examined whether the dissociating effects of YIAD001 and YIAD002 *in vitro* could be translated to the dissociation and clearance of amyloid plaques in the brains of 5XFAD mice, a transgenic mouse model of amyloid pathology [18]. During a 5-week timespan, 4.5-month-old female 5XFAD mice were administered YIAD001 (50 mg/kg/day, *n* = 6) and YIAD002 (50 mg/kg/day, *n* = 6) through drinking water. Vehicle-treated littermate 5XFAD mice (*n* = 7) and wildtype mice (*n* = 7) were used as controls. Furthermore, mice were treated with an inactive compound, YIAD003 (50 mg/kg/day, *n* = 6), as an additional control. After administration, the mice were sacrificed at the age of 5.5 months, and their brains were immunostained with anti-Aβ 6E10 antibody to assess amyloid plaque burden (Fig. 1F, G). In comparison to vehicle-treated 5XFAD mice, the number of 6E10-stained plaques were significantly reduced in mice treated with YIAD001 (*P* < 0.05) and YIAD002 (*P* < 0.001), while YIAD003 did not cause any change in plaque count (Fig. 1H). Upon quantifying plaque area, we found that only YIAD002 led to a significant decrease (*P* < 0.05), whereas a slight tendency of an increase was observed in the group treated with YIAD003 (Fig. 1H).

Followingly, soluble Aβ levels in the cortex and hippocampus were assessed for the groups with lowered amyloid burden. Dot blot assays were performed using anti-Aβ 6E10 antibody to detect total levels of Aβ and A11 antibody to detect oligomeric Aβ species. In cortical lysates, YIAD001 and YIAD002 administration significantly reduced the levels of 6E10-detected total Aβ (*P* < 0.01 and *P* < 0.05) and exhibited a trend of reduction in A11-detected oligomer levels (*P* = 0.1086 and *P* = 0.2486) (Fig. 1I), suggesting the clearance of Aβ from the brain after plaque dissociation. In the hippocampus, 6E10-detected levels of total Aβ were not significantly changed; however, oligomeric Aβ content was significantly reduced by YIAD002 (*P* < 0.01) (Fig. 1J). Since 6E10 antibody is also known to capture APP, we additionally examined the changes in cortical and hippocampal APP levels via western blot analysis (Fig. 1K, L). We confirmed that YIAD001 and YIAD002 did not induce significant shift in overall amounts of APP, proposing that the preceding dot blot results predominantly reflect changes in Aβ levels.

To further confirm the reduction of total Aβ levels in the soluble fractions of brain lysates, dot blot assay with 6E10 antibody was once again performed after removing APP from the sample. Strong denaturant GdnHCl was first utilized to further solubilize and denature proteins in cortical lysates. The samples were then filtered utilizing a 100 kDa MWCO filter to remove APP (100–140 kDa), and the subsequent dot blot results exhibited reduction of Aβ in YIAD001 and YIAD002 groups, reaffirming the earlier dot blot analysis to account for the amount of total Aβ levels.

### YIAD002 attenuates Aβ-associated pathology in 5XFAD mice

**Fig. 2:**
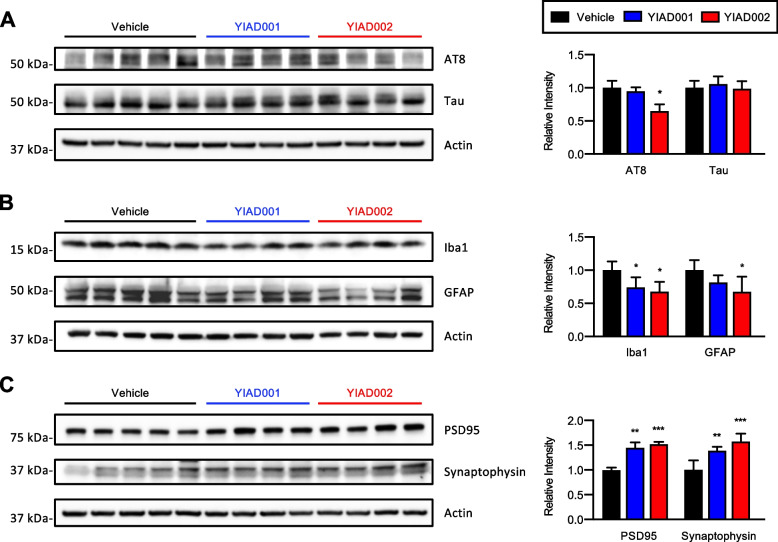
YIAD001 and YIAD002 ameliorates the expression levels of AD-related biomarkers. YIAD001 (50 mg/kg/day, *n* = 6) and YIAD002 (50 mg/kg/day, *n* = 6) were orally administered to 4.5-month-old female 5XFAD mice for 5 weeks. Through western blot, soluble fractions of cortical lysates were immunoblotted for AT8 and tau (**A**), Iba1 and GFAP (**B**), PSD95 and synaptophysin (**C**), and actin (**A–C**). Relative intensities of the blots were quantified in ratio to actin and statistically compared to vehicle-treated 5XFAD mice (Vehicle). Full blot images are provided in additional files. One-way analysis of variance followed by Bonferroni’s post hoc comparisons tests were performed in all statistical analyses (*P<0.05, **P<0.01, ***P<0,001, ****P<0.0001). Data are presented as mean ± SEM

Next, we investigated whether the reduction of Aβ aggregates in the 5XFAD mouse brain by YIAD001 and YIAD002 could attenuate major pathological hallmarks of AD. As studies show that tau pathology can be reduced by Aβ clearance in transgenic AD mice and AD patients [19], we tested whether chemical-driven Aβ disaggregation by YIAD compounds affected tauopathy in 5XFAD mice. Although 5XFAD mice are amyloid deposition models, detectable levels of phosphorylated tau have been reported [20], and thus, we immunoblotted the cortical lysates of 5XFAD mice with AT8 antibodies, which react with tau phosphorylated at Ser202 and Thr205, and anti-tau antibodies. We found that while YIAD001 did not affect cortical phosphorylated tau levels, YIAD002 significantly reduced phosphorylated tau by 35.29% (*P* < 0.05), while cortical total tau levels were not affected by either compound (Fig. 2A).

Microgliosis and reactive astrogliosis are key markers of neuroinflammation characteristic of AD pathology [21]. To examine whether the disaggregation of amyloid plaques by YIAD001 and YIAD002 administration attenuates Aβ-induced gliosis, brain lysates of 5XFAD mice were immunoblotted with Iba1 and GFAP antibodies to assess microgliosis and astrogliosis, respectively. In comparison to the vehicle-treated mice, cortical Iba1 levels were decreased in mice administered YIAD001 (26.08% reduction, *P* < 0.05) and YIAD002 (32.11% reduction, *P* < 0.05) (Fig. 2B). GFAP expression was only significantly reduced in YIAD002-administered mice (32.76% reduction, *P* < 0.05) (Fig. 2B).

Increased Aβ levels, tauopathy, and neuroinflammation are reported as causative agents of synaptic dysfunction [22], and postsynaptic marker PSD95 and presynaptic marker synaptophysin are both reported to be downregulated in the presence of aggregated amyloid proteins [23]. We found that YIAD001 increased cortical levels of PSD95 by 44.59% (*P* < 0.01), while YIAD002 upregulated PSD95 by 51.97% in the cortex (*P* < 0.001) (Fig. 2C). Cortical levels of synaptophysin were increased by 38.68% in YIAD001-treated mice (*P* < 0.01) and 57.47% in YIAD002-treated mice (*P* < 0.001) (Fig. 2C).

Alteration of hippocampal protein markers were also examined, but did not exhibit the pharmaceutical effect observed in the cortical lysates (Fig. S2). As controls to assess alleviation of pathology, pathological marker alterations were assessed in age-matched wildtype mice using cortical and hippocampal lysates (Fig. S3). Comparative blots including wildtype mice and YIAD003 were also performed using cortical tissue (Fig. S4).

Overall, while both YIAD001 and YIAD002 strongly dissociated Aβ in vitro and considerably reduced amyloid burden in 5XFAD mice (Fig. 1), YIAD002 was shown to be more effective in alleviating downstream AD pathology, including tauopathy, neuroinflammation, and synaptic loss, in the cortex. As such, YIAD002 was selected for further characterization studies.

### BBB-penetration and ADME assessment of YIAD002

**Fig. 3:**
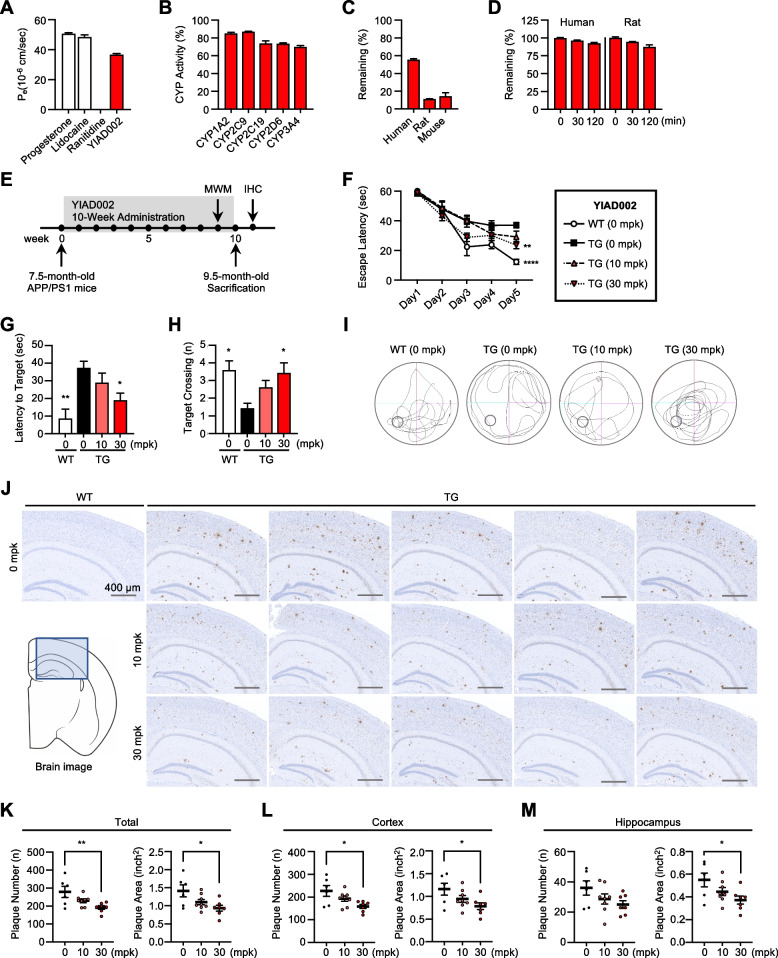
Assessment of druggability and in vivo replicability of YIAD002 in APP/PS1 mice. **A** Effective permeability coefficient (*P*_e_, 10^−6^ cm/s) of YIAD002 was estimated using BBB-PAMPA. **B** CYP inhibition profile. **C** Stability of YIAD002 in liver microsomes of human, rat, and mouse. **D** Stability of YIAD002 in human and rat plasma. **E** Scheme for APP/PS1 administration. YIAD002 (10 mg/kg/day, *n* = 8, and 30 mg/kg/day, *n* = 8) was orally administered to 7.5-month-old male APP/PS1 mice for 10 weeks. **F** Escape latency during Morris water maze. **G** Latency to target and **H** target crossings. **I** Representative traces of probe trial. **J** Images of 6E10-immunostained brains (scale bars: 400 μM). Plaque number and area of the whole brain (**K**), cortex (**L**), and hippocampus (**M**) of the mice were quantified. All statistical comparisons were made with the vehicle-treated APP/PS1 mouse group (TG (0 mpk)). One-way analysis of variance followed by Bonferroni’s post hoc comparisons tests were performed in all statistical analyses (*P < 0.05, **P < 0.01, ****P < 0.0001). Data are presented as mean ± SEM

To evaluate BBB permeability of YIAD002, we performed BBB-specific PAMPA to find the effective permeability coefficient (*P*_e_, 10^−6^ cm/s) [24]. BBB permeability can be classified into the following ranges: “high BBB permeation” (*P*_e_ > 4.0), “low BBB permeation” (*P*_e_ < 2.0), and “BBB permeation uncertain” (2.0 < *P*_e_ < 4.0) [25]. The *P*_e_ of high BBB permeation controls, progesterone (50 μM) and lidocaine (50 μM), were 50.85 10^−6^ cm/s and 48.46 10^−6^ cm/s, respectively, and the *P*_e_ of low BBB permeation control ranitidine (50 μM) was 0 10^−6^ cm/s. YIAD002 (50 μM) demonstrated high BBB permeability, with a *P*_e_ of 36.83 10^−6^ cm/s (Fig. 3A).

Next, we performed in vitro ADME assessments such as the CYP450 inhibition assay, hepatic microsome stability assay, and plasma stability assay. We assessed the alterations of CYP activity by YIAD002 (10 μM) on the five primary drug metabolizing CYPs, 1A2, 2C9, 2C19, 2D6, and 3A4, and found that YIAD002 had weak or no inhibition according to inhibition criteria (IC_50_ > 10 μM) (Fig. 3B). Next, the metabolic stability of YIAD002 in liver microsomes was examined to predict in vivo drug clearance [26]. Microsome solutions of human, rat, and mouse were treated with YIAD002 (1 μM) and the remaining compound was measured after 30 min. YIAD002 exhibited moderate stability in human microsomes (55.5%) and faster rates of metabolic turnover in rat (11.2%) and mouse (14.2%) microsomes (Fig. 3C). To investigate the potential degradation and modification by enzymes in plasma [27], we assessed compound plasma stability by incubating YIAD002 (10 μM) in human or rat plasma at 37°C for 0, 30, and 120 min. In comparison to YIAD002 concentration at 0 min, 96.3% and 92.6% of the compound were remaining at 30 and 120 min, respectively, in the supernatant of human plasma (Fig. 3D). Furthermore, 94.5% and 87.7% of YIAD002 was remaining at 30 and 120 min, respectively, in the rat plasma supernatant (Fig. 3D).

### YIAD002 improves learning and memory in APP/PS1 mice

To confirm the in vivo efficacy of YIAD002 against Aβ, we tested if administration of YIAD002 could improve cognitive functions and reduce Aβ burden in another AD mouse model, the APP/PS1 transgenic mouse (Fig. 3E). YIAD002 was orally administered at two dosages, 10 mg/kg/day (*n* = 8) and 30 mg/kg/day (*n* = 7), for 10 weeks to 7.5-month-old APP/PS1 mice. Vehicle-treated wildtype (*n* = 5) and APP/PS1 (*n* = 6) littermates were used as controls. Learning and memory were evaluated through the Morris water maze test during the tenth week of drug administration. After 5 days of training, escape latency was significantly shortened in the wildtype group (*P* < 0.0001) and APP/PS1 group treated with 30 mg/kg/day of YIAD002 (*P* < 0.01) in comparison to vehicle-treated APP/PS1 mice (Fig. 3F). During the probe trial, mice administrated YIAD002 (30 mg/kg/day) displayed significantly shorter latency to target (*P* < 0.05) and increased target crossings (*P* < 0.05) (Fig. 3G–I). After the end of administration, we performed immunohistochemical staining on the brains of APP/PS1 mice with anti-Aβ 6E10 antibody to assess the alteration of amyloid plaque burden (Fig. 3J). As a result, the number and area of 6E10-stained plaques were reduced throughout the whole brain of YIAD002-treated mice in comparison to those of vehicle-treated mice (*P* < 0.01 and *P* < 0.05) (Fig. 3K). Furthermore, YIAD002 significantly reduced the number and size of plaques (*P* < 0.05 and *P* < 0.05) in the cortex (Fig. 3L) and plaque size in the hippocampus (*P* < 0.05) of APP/PS1 mice (Fig. 3M).

### Peptide mapping and docking simulations suggest dissociation sites

**Fig. 4:**
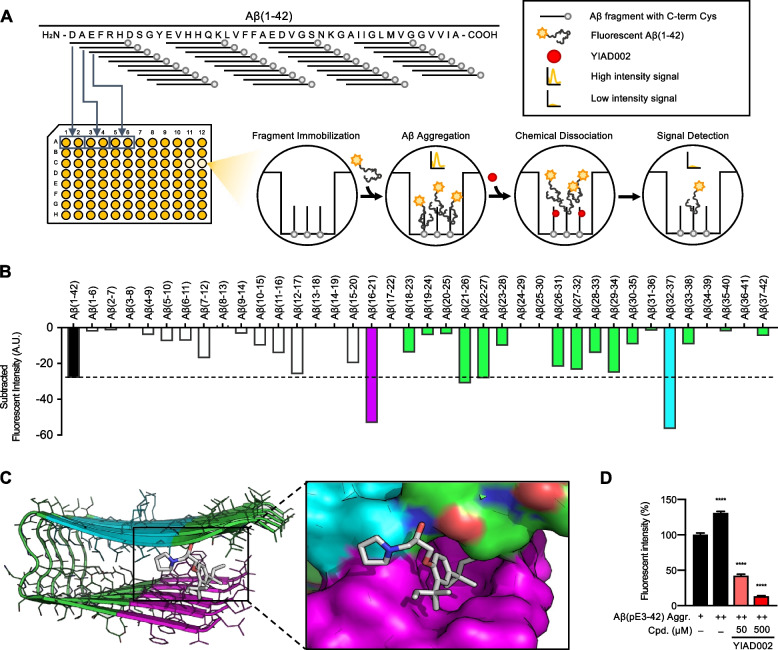
YIAD002 disrupts Aβ aggregation by blocking intermolecular folding of paired β-strands. **A** Scheme of mapping assay using partial fragments of Aβ. **B** Mapping assay indicates region of Aβ dissociation by YIAD002: Aβ(16–21) in purple and Aβ(32–37) in cyan. **C** Docking models of YIAD002 interaction with Aβ(1–42). **D** Dissociative effects of YIAD002 against Aβ(pE3-42) aggregation were evaluated using ThT fluorescence. Aggregation for Aβ(pE3-42) is indicated as “+” for 1 day, and “++” for 4 days. Fluorescence intensities were normalized and statistically compared to the Aβ(pE3-42)-only 1 day (+) control (100%). One-way analysis of variance followed by Bonferroni’s post hoc comparisons tests were performed in all statistical analyses (****P < 0.0001). Data are presented as mean ± SEM. Conc. = concentration, Aggr. = aggregation, Cpd. = compound

To elucidate the sequence-specific sites of interaction between YIAD002 and Aβ, we fabricated a mapping assay using peptide fragments of Aβ(1–42) (Fig. 4A). Overlapping Aβ fragments consisting of 6 amino acid residues and a cysteine residue at the C-terminal were synthesized and immobilized onto a maleimide-activated microplate. To induce Aβ-Aβ formations, we added monomeric Aβ(1–42) (10 μM) labeled with fluorescent dye Flamma 552 to each well. After incubating the plate for 2 h at 37°C, subsequent Aβ-Aβ formations were detectable by fluorescence intensity, establishing a fluorescence baseline for each well. YIAD002 (500 μM) was treated to each well and additionally incubated for 24 h at 37°C, and reduction of fluorescence intensity in individual wells was quantified (Fig. 4B). When compared to the Aβ(1–42) control, in which full-length Aβ(1–42) was attached to the well, we found that YIAD002 markedly dissociated Aβ-Aβ interactions at residues Aβ(16–21), KLVFFA, and Aβ(32–37), IGLMVG, which are both recognized aggregation-prone hydrophobic sequences of Aβ. Loss of hindrance in fragmented Aβ in comparison to full length Aβ may allow higher accessibility for YIAD002 to interact and dissociate aggregates. The dissociation peaks are not perfect representations of sequence-specific interactions, as there is a possibility that reduced signals are partially from the dissociation of additional aggregates composed of Flamma 552-labeled Aβ(1–42), so data should be interpreted in comparison to the Aβ(1–42) control.

Using the molecular structure of YIAD002, we performed constrained docking simulations to elucidate target-ligand binding conformation inducing the dissociation of Aβ aggregates. To consider the structural polymorphism of Aβ oligomers, various Aβ(1–42) aggregate structures in the Protein Data Bank (PDB) were all considered for docking simulation (Table S1). Sequences KLVFFA and IGLMVG were used to confine binding sites of multiple YIAD002-Aβ conformers. The docking model with the lowest binding energy of −7.0 kcal/mol, a β-strand-turn-β-strand motif containing intermolecular β-sheets of Aβ (PDB ID 2BEG), was used for the following structural analysis. Parallel β-sheets are formed through intermolecular side-chain contacts between residues 18–26 of one Aβ and the residues 31–42 of the next Aβ. Our docking model showed that YIAD002 is located between β-sheet assemblies, simultaneously interacting with Aβ(16–21), KLVFFA, and Aβ(32–37), IGLMVG (Fig. 4C). The hydrophobic group around the aromatic ring in YIAD002 is tightly packed into the cleft formed by the repetitive array of Leu17 and Phe19 residues. On the other hand, the pyrrolidine group on the opposite side of the hydrophobic group in YIAD002 contacts the backbone of Val36 and Gly37 of the paired β-strand. As the docking pose is shown to effectively block intermolecular β-strand pairing, our results suggest that YIAD002 dissociates Aβ aggregation by disrupting and inhibiting further β-sheet fibrillation.

### YIAD002 binds to and reduces aggregation of tau fragments

**Fig. 5:**
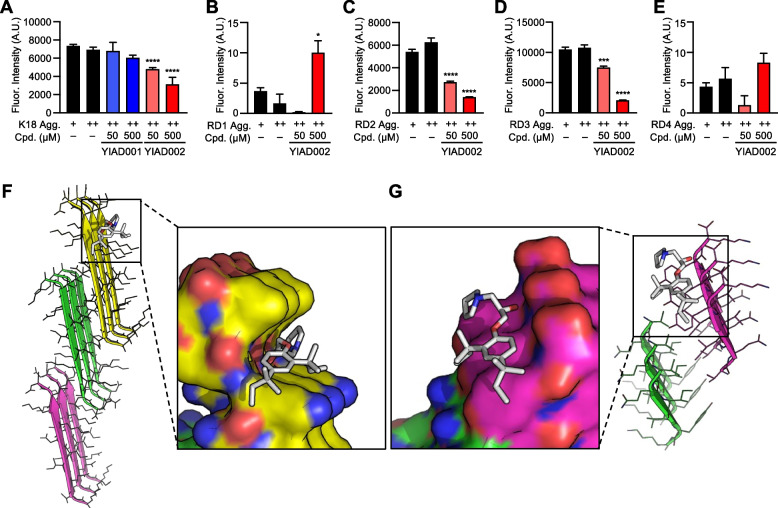
YIAD002 moderately dissociates tau aggregation by interacting with R2 and R3. **A** Dissociative effects of YIAD001 and YIAD002 against K18 aggregation were evaluated using ThT fluorescence. Dissociative interactions by YIAD002 with tau fragments R1 (**B**), R2 (**C**), R3 (**D**), and R4 (**E**) were evaluated by ThT fluorescence. Docking models of YIAD002 interaction with tau R2 (**F**) and R3 (**G**). Aggregation times are indicated as “+” for 3 days, and “++” for 6 days. All statistical comparisons were made with the pre-aggregation control (+). One-way analysis of variance followed by Bonferroni’s post hoc comparisons tests were performed in all statistical analyses (*P < 0.05, ***P < 0.001, ****P < 0.0001). Data are presented as mean ± SEM. Abbreviations: Conc. = concentration, Agg. = aggregation, Cpd. = compound, Fluor. = fluorescent

YIAD002 had better therapeutic efficacy against downstream AD pathology in comparison to YIAD001, despite having similar dissociating effects against Aβ burden in vivo (Fig. 2). As the aggregation of tau into paired helical filaments is also driven by β-sheet fibrillation [28], we hypothesized that YIAD002 may have additional inhibitory functions against tau aggregation, providing stronger resistance to AD pathology progression than YIAD001 in vivo. To test this hypothesis, we examined the dissociating effect of YIAD001 and YIAD002 against K18, a recombinant fragment of tau comprising microtubule-binding domain, the critical region driving β-sheet formation [28]. After forming aggregates of K18 (35 μM) by incubating the peptides at 37°C for 3 days, 50 and 500 μM of YIAD001 and YIAD002 were co-incubated with pre-aggregated K18 for an additional 3 days. Through ThT fluorescence assay, we found that YIAD002 significantly reduced K18 aggregates by 58.96% (*P* < 0.001), while YIAD001 did not have a significant effect (18.06% disaggregation, *P* = 0.4224) (Fig. 5A).

The microtubule-binding domain of K18 comprises four repeats R1, R2, R3, and R4 [29]. To locate which repeats YIAD002 interacts with, we performed additional ThT assays on the four individually synthesized repeats. Individual repeats were pre-incubated at 37°C for 1 day and YIAD002 (50 and 500 μM) was co-incubated for an additional 3 days. We found that while β-sheet formation of R1 and R4 were not detectable through ThT, R2 and R3 could sufficiently form β-sheet fibrils that were subsequently dissociated by YIAD002 (R2: 73.99% disaggregation, *P* < 0.0001; RD3: 80.38% disaggregation, *P* < 0.0001) (Fig. 5B–E).

As YIAD002 was shown to reduce β-sheet-rich aggregates of R2 and R3, docking simulations for tau were performed for both repeat sequences. For R2, docking simulations were performed identically on two different oligomeric R2 structures (5V5B and 5V5C), and the docking model with the lowest binding energy (−6.3 kcal/mol for 5V5B) was used for the structural analysis. The docking model suggested that YIAD002 blocks interface formation by binding the cleft between Lys280 and Leu282 of tau β-sheet structures, and the hydrophobic group attached to the aromatic ring in YIAD002 is positioned next to the alkyl-chain of Lys280 (Fig. 5F). These results suggest that the docking of YIAD002 prevents further aggregation of R2 tau oligomers. Similar results were also shown for the docking model with 5V5C (Table S1). The sequence segment VQIVYK (PDB ID 2ON9) was used as a receptor structure for R3 and the predicted docking score of YIAD002 was −5.3 kcal/mol. Structural analysis of the docking model suggested that YIAD002 blocks interface formation by binding the cleft between Tyr310 and Lys311 of R3 oligomer β-sheet structures (Fig. 5G). The hydrophobic group attached to the aromatic ring in YIAD002 is positioned next to the aromatic ring of Tyr310, possibly preventing the further aggregation of tau oligomers. Overall, the docking studies consistently showed that YIAD002 dissociates aggregates of Aβ and tau by interacting with the clefts formed between successive placements of hydrophobic residues in β-sheet structures. To examine whether YIAD002 interacts with other amyloidogenic proteins with β-sheet secondary structures, we performed an additional dissociation assay against α-synuclein, a β-sheet-rich protein implicated in Parkinson’s disease [30], and found that YIAD002 did not interact with α-synuclein aggregates (Fig. S 4).

## Discussion

Here, we report the discovery of aryloxypropanolamine derivative YIAD002 that strongly dissociates Aβ aggregates in vitro and in vivo, by interacting with paired intermolecular β-strand structures contributing to β-sheet aggregation. We first identified the dissociative activity of YIAD002 against Aβ aggregation and confirmed biochemical activity in transgenic AD mice by observing amyloid clearance and concomitant cognitive enhancement and amelioration of downstream pathology. Fragment peptide mappings and constrained docking simulations indicate that YIAD002 directly dissociates Aβ by interacting with hydrophobic clefts in intermolecular β-sheet structures. Furthermore, we discovered that YIAD002 has moderate dissociative activity against tau aggregates by interacting with the microtubule-binding repeat domain driving β-sheet formation.

Mapping assay using Aβ fragments revealed that YIAD002 mainly dissociates Aβ-Aβ interaction at Aβ(16–21), KLVFFA, and Aβ(32–37), IGLMVG. KLVFFA is reported as the most aggregation-prone portion of Aβ, located at the central hydrophobic core of amyloid fibrils [31]. Solid state NMR studies indicate that β-sheets of Aβ fibrils are held together by internal quaternary contacts between Leu17 and Phe19 residues of KLVFFA with Ile32, Leu34, and Val36 of IGLMVG [32]. Our molecular docking simulations show that YIAD002 localizes within clefts formed between intermolecular KLVFFA and IGLMVG, with hydrophobic groups around the aromatic ring packed in between Leu17 and Phe19 and the pyrrolidine group contacting the backbone of Val36 and Gly37. Connecting these results to the initial 11 1-amino-3-phenoxypropan-2-ol derivatives screened for inhibitory interaction with Aβ(1–42), compounds with alkylbenzene groups and pyrrolidine groups had higher inhibitory activity against Aβ(1–42) aggregation, suggesting potential structure-activity relationship.

Although YIAD001 and YIAD002 both significantly decreased amyloid burden in 5XFAD mice, YIAD002 exhibited superior efficacy against hyperphosphorylated tau, gliosis, and synaptic protein loss. Through ThT fluorescence assays and docking simulations, we found YIAD002 possessed additional dissociative activity against tau aggregation by interacting with β-sheet-driving regions R2 and R3. These results suggest that the dual targeting of Aβ and tau may be beneficial in halting pathological cascades leading to synaptic dysfunction and cognitive decline. Several preclinical studies have exemplified the synergistic therapeutic effects exerted by the dual targeting of aggregated Aβ and tau through improved cognitive behavior in transgenic AD mice [22, 33].

## Limitations for this study

Our study has some limitations. Although we predict that YIAD002 interacts with β-sheet formations involving KLVFFA and IGLMVG through mapping assays and constrained molecular docking, Aβ aggregates exist as transient and polymorphic structures. Our results predicted interactions between YIAD002 and U-shaped protofibrils and cannot represent interactions with all aggregates of Aβ.

Furthermore, adverse effects of toxicity by YIAD002 were not observed in our current studies, additional investigations of off-target interactions and in vivo toxicological studies of higher doses are needed prior to further clinical advancement.

## Conclusions

In summary, our results demonstrate the therapeutic potential of YIAD002, a strong dissociator of Aβ and tau aggregation. Following the recent FDA approval of aducanumab as the first disease-modifying treatment for AD [34], we can anticipate the appropriate next step as the development of chemical alternatives capable of dissociation and clearance of Aβ aggregates. In concurrence with current literature, our study provides supporting evidence that amyloid disaggregation by small molecules can modify downstream pathology and enhance cognitive function.

## Supplementary Information

**Additional file 1: Figure S1.** Dot blot assay of A11-detected amyloid oligomers to assess *in vitro* disaggregation of Aβ(1-42) by YIAD001 and YIAD002. **Figure S2.** Western blot and relative densitometries of hippcampal lysates of control and drug administered 5XFAD mice. **Figure S3.** Comparative western blot analysis of control wildtype and 5XFAD mice. **Figure S4.** Western blot and relative densitometries of cortical lysates of controls and drug administered 5XFAD mice. **Figure S5.** ThT dissocation assay against α-synuclein aggregation. **Table S1.** Polymorphic structures of Aβ and tau used for constrained docking simulations.

## Data Availability

The datasets used and/or analyzed during the current study are available from the corresponding author on reasonable request.

## Abbreviations

Aβ: Amyloid-β
AD: Alzheimer’s disease
APP: Amyloid precursor protein
BBB: Blood-brain barrier
ThT: Thioflavin T
PAMPA: Parallel artificial membrane permeability assay
R1: Repeat 1
R2: Repeat 2
R3: Repeat 3
R4: Repeat 4
PICUP: Photo-induced cross-linking of unmodified proteins
GdnHCl: Guanidinium hydrochloride
MWCO: Molecular weight cut-off
CYP450: Cytochrome P450s
IACUC: Institutional Animal Care and Use Committee
Iba1: Ionized calcium-binding adapter molecule
GFAP: Glial fibrillar acidic protein
PSD95: Postsynaptic density protein 95
PDB: Protein Data Bank
