# Supplementary material for: Aryloxypropanolamine targets amyloid aggregates and reverses Alzheimer-like phenotypes in Alzheimer mouse models
Source: Alzheimers Res Ther. 2022 Nov 29;14:177. doi: 10.1186/s13195-022-01112-6 (PMC9706920; doi:10.1186/s13195-022-01112-6)
Supplement: Supplementary file 1 — Additional file 1: Figure S1. Dot blot assay of A11-detected amyloid oligomers to assess in vitro disaggregation of Aβ(1-42) by YIAD001 and YIAD002. Figure S2. Western blot and relative densitometries of hippcampal lysates of control and drug administered 5XFAD mice. Figure S3. Comparative western blot analysis of control wildtype and 5XFAD mice. Figure S4. Western blot and relative densitometries of cortical lysates of controls and drug administered 5XFAD mice. Figure S5. ThT dissocation assay against α-synuclein aggregation. Table S1. Polymorphic structures of Aβ and tau used for constrained docking simulations. [file 13195_2022_1112_MOESM1_ESM.docx]

**Supplementary Information**

Title: Aryloxypropanolamine targets amyloid aggregates and reverses Alzheimer-like phenotypes in Alzheimer mouse models

**Authors:** HeeYang Lee^†^, Soljee Yoon^†^, Jeong Hwa Lee^†^, Keunwan Park, Youngeun Jung, Illhwan Cho, Donghee Lee, Jisu Shin, Kyeonghwan Kim, Sunmi Kim, Jimin Kim, Koeun Kim, Seung Hoon Han, Seong Muk Kim, Hye Ju Kim, Hye Yun Kim, Ikyon Kim^,*^, and YoungSoo Kim^*^


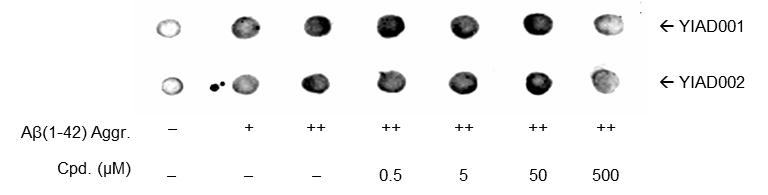


**Figure S1.** **Dot blot assay of A11-detected amyloid oligomers to assess *in vitro* disaggregation of Aβ(1-42) by YIAD001 and YIAD002.** Aggregation time for Aβ(1-42) is indicated as ‘–’ for 0 day, ‘+’ for 3 days, and ‘++’ for 6 days.


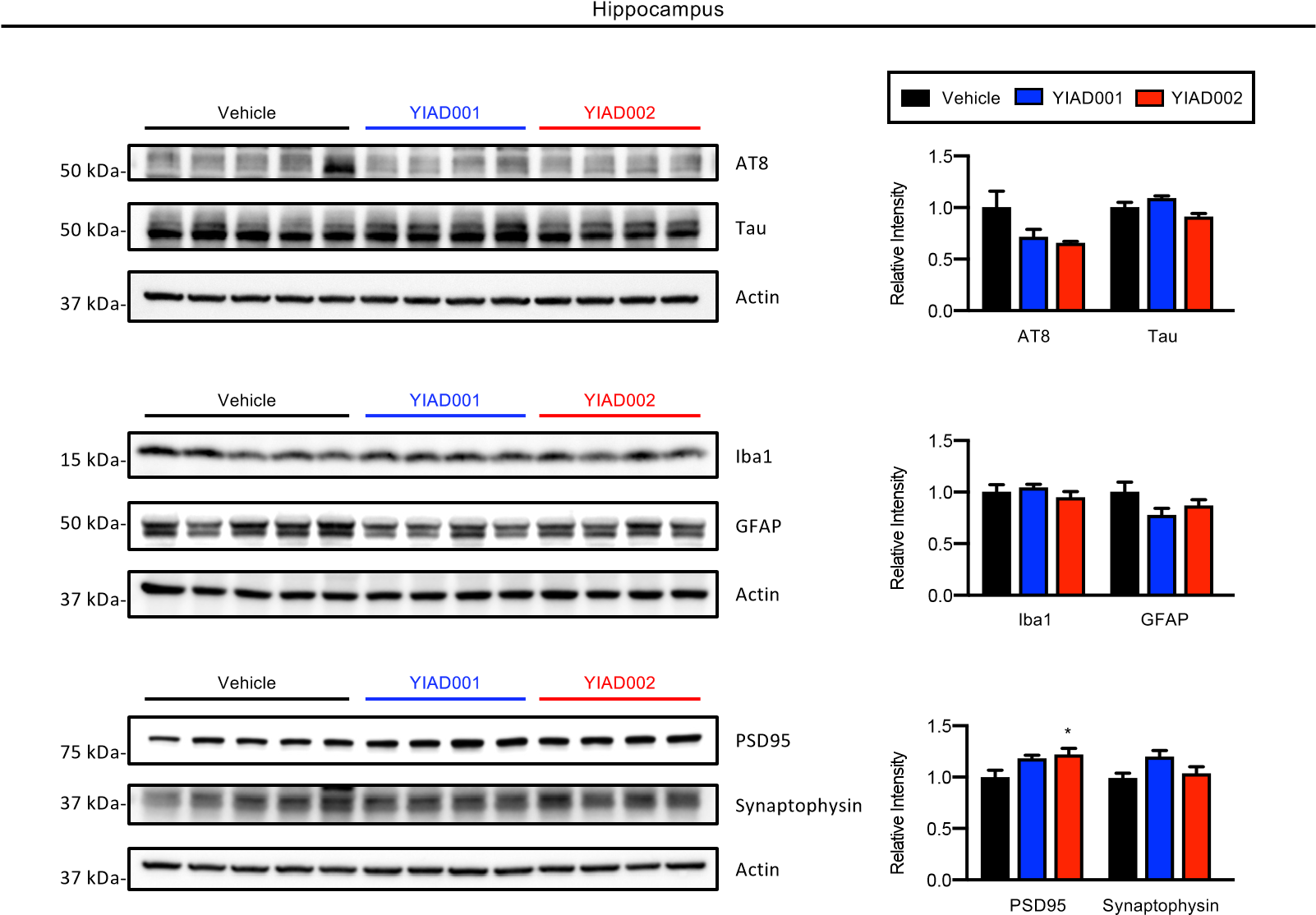


**Figure S2.** **Western blot and relative densitometries of hippcampal lysates of control and drug administered 5XFAD mice.** Through western blot, soluble fractions of hippocampal lysates were immunoblotted for AT8, tau, Iba1, GFAP, PSD95, synaptophysin, and actin. Relative intensities of the blots were quantified in ratio to actin and statistically compared to vehicle-treated 5XFAD mice (Vehicle). One-way analysis of variance followed by Bonferroni’s post-hoc comparisons tests were performed in all statistical analyses (*P < 0.05, **P < 0.01, ***P < 0.001, ****P < 0.0001). Data are presented as mean ± SEM.

**Figure S3.** **Comparative western blot analysis of control wildtype and 5XFAD mice.** Wildtype mice (n=7) and vehicle-treated 5.5-month-old 5XFAD mice (n=7) were used as controls for YIAD001 and YIAD002. Cortical and hippocampal lysates were immunoblotted for APP, Iba1, GFAP, AT8, tau, PSD95, synaptophysin, and actin. Relative intensities of the blots were quantified in ratio to actin and statistically compared to vehicle-treated 5XFAD mice (Vehicle). Full blot images are provided in additional files. One-way analysis of variance followed by Bonferroni’s post-hoc comparisons tests were performed in all statistical analyses (*P<0.05, **P<0.01, ***P<0,001, ****P<0.0001). Data are presented as mean ± SEM.


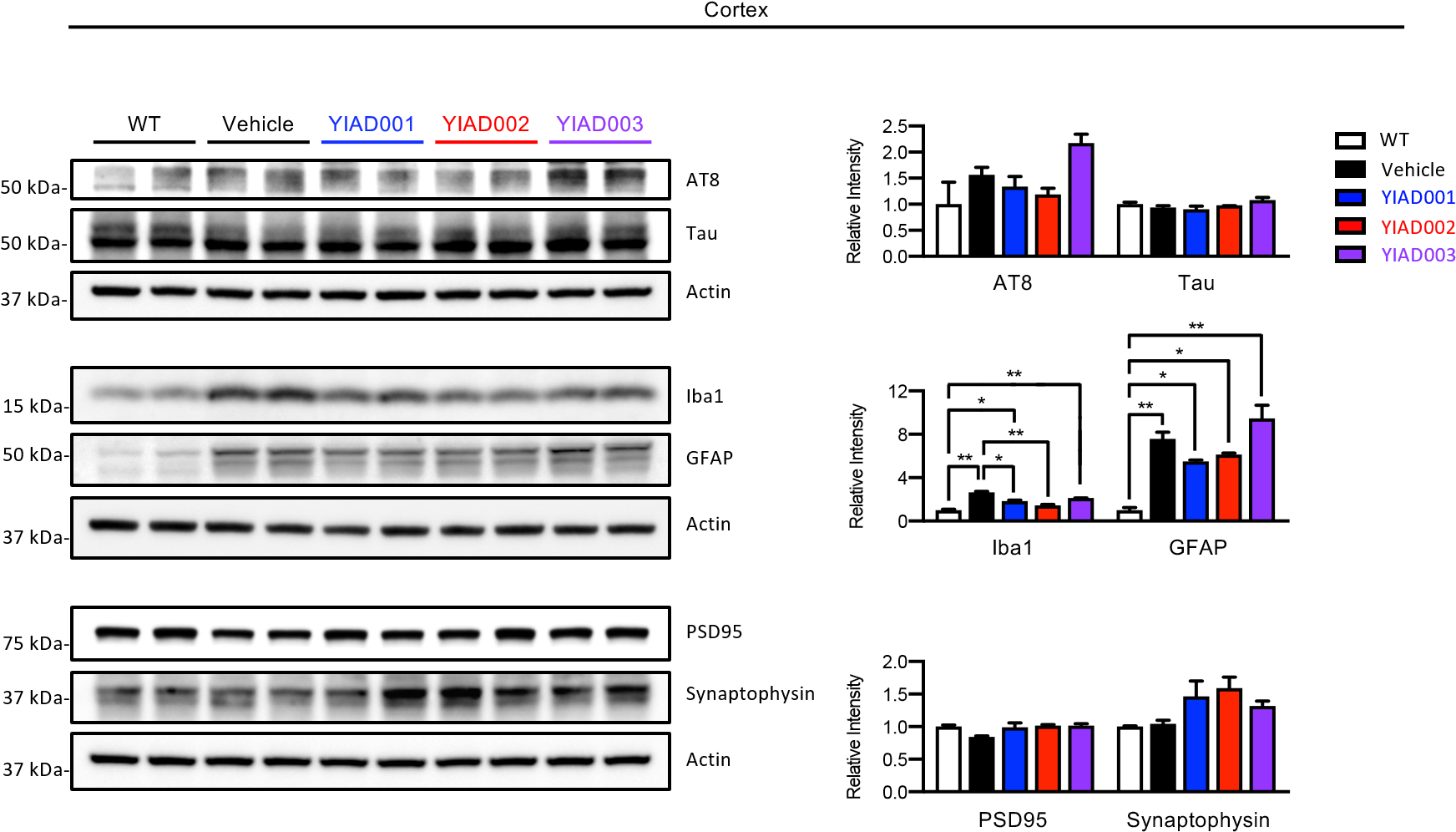


**Figure S4.** **Western blot and relative densitometries of cortical lysates of controls and drug administered 5XFAD mice.** YIAD001 (50 mg/kg/day, n = 6), YIAD002 (50 mg/kg/day, n = 6), and YIAD003 (50 mg/kg/day, n = 6) were orally administered to 4.5-month-old female 5XFAD mice for five weeks. Vehicle-treated 5XFAD mice (n = 7) and wildtype mice (n = 7) were used as controls. Through western blot, soluble fractions of cortical lysates were immunoblotted for AT8, tau, Iba1, GFAP, PSD95, synaptophysin, and actin. Relative intensities of the blots were quantified in ratio to actin and statistically compared between groups. One-way analysis of variance followed by Bonferroni’s post-hoc comparisons tests were performed in all statistical analyses (*P < 0.05, **P < 0.01, ***P < 0.001, ****P < 0.0001). Data are presented as mean ± SEM.


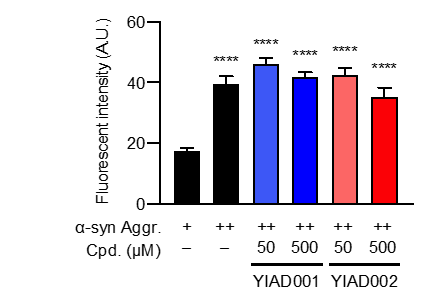
**Figure S5. ThT dissocation assay against α-synuclein aggregation.** Dissociative effects of **YIAD001** and **YIAD002** against α-synuclein aggregation were evaluated using ThT fluorescence. α-synuclein (35 μM) was pre-aggregated for 3 days. YIAD001 and YIAD002 were added to and co-incubated with pre-aggregated samples for an additional 3 days. Aggregation time for α-synuclein is indicated as ‘+’ for 3 days and ‘++’ for 6 days. All fluorescence intensities were normalized and statistically compared to α-synuclein-only 3 days (+) control (100%). One-way analysis of variance followed by Bonferroni’s post-hoc comparisons tests were performed in all statistical analyses (*****P* < 0.0001). Data are presented as mean ± SEM. Abbreviations: Aggr. = Aggregation, Cpd. = Compound.

**Table S1.** **Polymorphic structures of Aβ and tau used for constrained docking simulations.**

|  | Structure^[a]^ | PDB ID | Motif | Symmetry | Docking Score[b]  (kcal/mol) | Structure Reference |
| --- | --- | --- | --- | --- | --- | --- |
| Aβ (1-42) | 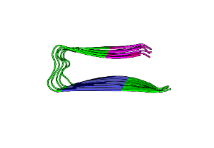 | 2BEG | U-shaped | Single-fold | -7.0 | (2005) Proc Natl Acad Sci U S A **102**: 17342-17347 |
|  | 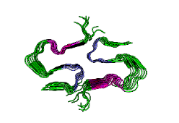 | 5KK3 | S-shaped | Two-fold | -5.9 | (2016) J Am Chem Soc **138**: 9663-9674 |
|  | 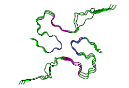 | 2NAO | S-shaped | Two-fold | -5.9 | (2016) Proc Natl Acad Sci U S A **113**: E4976-E4984 |
|  | 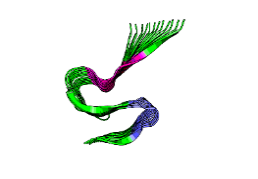 | 2MXU | S-shaped | Single-fold | -5.5 | (2015) Nat Struct Mol Biol **22**: 499-505 |
| Tau | 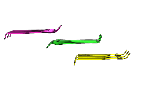 | 5V5B | 10-mer  (KVQIINKKLD) | Three-fold | -6.3 | (2018) Nat Chem **10**: 170-176 |
|  | 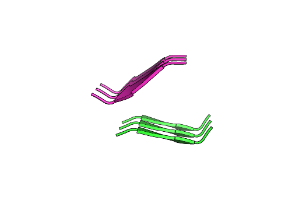 | 5V5C | 6-mer  (VQIINK) | Two-fold | -5.2 | (2018) Nat Chem **10**: 170-176 |

[a] In Aβ, magenta and blue represent Aβ(16-21) and Aβ(32-37), respectively. In Tau, colors are used to differentiate beta strands. [b] Docking score is predicted by Autodock vina.
